# Seroprevalence of hepatitis B and C virus infections among health students and health care workers in the Najran region, southwestern Saudi Arabia: The need for national guidelines for health students

**DOI:** 10.1186/1471-2458-14-577

**Published:** 2014-06-09

**Authors:** Jobran M Alqahtani, Saeed A Abu-Eshy, Ahmed A Mahfouz, Awad A El-Mekki, Ahmed M Asaad

**Affiliations:** 1 Department of Child Health, College of Medicine, Najran University, Najran, Saudi Arabia; 2 Department of Surgery, College of Medicine, Najran University, Najran, Saudi Arabia; 3 Department of Family and Community Medicine, College of Medicine, King Khalid University, Abha, P.O. Box 641, Saudi Arabia; 4 Department of Clinical Microbiology, College of Medicine, King Khalid University, Abha, Saudi Arabia; 5 Departments of Microbiology, College of Medicine, Najran University, Najran, Saudi Arabia

**Keywords:** HBV, HCV, HCW, Health Students, Saudi Arabia

## Abstract

**Background:**

The objectives of the study were to study the seroprevalence of hepatitis B virus (HBV) and hepatitis C virus (HCV) infections among health college students (HS) and health care workers (HCWs) in the Najran Region of south-western Saudi Arabia and to study the students’ knowledge of occupational exposure to blood-borne viral infections.

**Methods:**

A cross-sectional study of a representative sample of 300 HS and 300 HCWs was conducted.

**Results:**

An overall seroprevalence of HBV of 1.7% and 8.7% was found among HS and HCWs, respectively. Two-thirds of HS (66.7%, 200) and 23.3% (70) of HCWs lack anti-HBs and are susceptible to HBV infection. An overall seroprevalence of HCV of 0% and 0.3% was found among the HS and HCWs, respectively. The present study indicates poor knowledge among HS and moderate knowledge among HCWs regarding occupationally transmitted blood-borne diseases, safe injection practices, and standard precautions to prevent occupationally transmitted blood-borne infections.

**Conclusion:**

It is mandatory to develop a structured program to raise awareness among HS, and current health colleges’ curricula should be upgraded to address these issues early. The HS should be considered new recruits to health services in terms of their initial screening for blood-borne infections and vaccination against HBV. The development of a novel continuing medical education and pre-employment awareness program for HCWs is recommended to address the following: blood-borne diseases transmitted occupationally, standard precautions to prevent occupationally transmitted blood borne infections, and safe injection practices.

## Background

Blood-borne infections have been recognised as an occupational hazard for nearly 50 years. However, it is only in the last 20 years that there has been a widespread recognition of the specific risk posed to health care workers by blood-borne viruses such as hepatitis B virus (HBV), hepatitis C virus (HCV) and human immunodeficiency virus (HIV).

Many studies have been published in the last two decades addressing various aspects of HBV infection in Saudi Arabia, such as its prevalence among the general population and the different age groups [1-5], blood donors [6,7], health care workers [8], pregnant women [9], virus genotypes and its relation to hepatocellular carcinoma [10,11]. These studies indicate that the disease is endemic in the kingdom and of major public health importance.

In 2003, a study [2] documented a high endemicity of HBV; 5% to 10% of the population were infected. The highest rate of infection was in the southern region of Saudi Arabia. By 1989, one year prior to the addition of HBV vaccine to the expanded programme of immunisation (EPI) in 1990, the prevalence among children was approximately 7% [2]. A steady decline in HBV infection among children was subsequently observed [12]. A recent study conducted in regions of different HBV endemicity in Saudi Arabia [13], documented a zero prevalence of HBsAg among students (16 – 18 years of age), which thus documents the efficacy of HBV vaccine and its long-term protection.

Hepatitis B vaccine is recommended for pre- and/or post-exposure prophylaxis of all persons at risk of contact with blood, blood products, or bodily secretions. Ideally, immunisation against hepatitis B should be completed prior to health professional training because the risk for infection is thought to be highest at this time. Immunising HCWs against hepatitis B prevents nosocomial transmission of the virus from HCWs to patients and from patient to HCWs [14].

Despite limitations in studies from various regions of the kingdom, Madani [15] reported a rate of 124 HCV cases per 100,000 population, or 0.124%, and observed a slight steady increase during the period 1995 – 2005, followed by a plateau. Furthermore, there were lower rates of infections among children compared to with adults, which suggests that perinatal and childhood transmission was not a major mode of transmission.

The Najran region is located in the southwest of Saudi Arabia along the border with neighbouring Yemen. It has an area of 360,000 km^2^. Its capital is Najran City. Recent updated data revealed that students currently enrolled in health colleges in Najran University amounted to more than nine hundred male and female students. Ministry of Health data revealed that in the Najran region, a total of six hundred male and female physicians and nurses are currently working in hospitals and primary health care centres.

The objectives of this work were to study the seroprevalence and the state of immunity to HBV infection and the rate of HCV infections among HCWs in the Najran region and HS in Najran University and to determine the degree of their knowledge, attitude and practice (KAP) of occupational exposure to blood and risk of blood-borne viral infections.

## Methods

### Settings

The study was a cross-sectional study of a representative sample of HCWs and HS in Najran Region, Southwestern Saudi Arabia.

### Sample size determination sampling procedures

Using the WHO manual for sample size determination in health studies [16], with a 95% confidence interval with a conservative estimate of the anticipated population proportion of 5% [2] and an absolute precision of 2%, the minimal sample size required for the study was calculated to be 457 persons. To avoid loss of cases, a total sample of 600 persons was planned to be included in the present study. A stratified proportional allocation random sample was selected. The stratification factors to be taken into consideration were the relative number of HCWs in hospitals and primary health care centres and students in each health college, male–female differences, and student’s level.

### Ethical approval

The study was reviewed and approved by the ethical committee of Najran University. Written consent was obtained from all participants.

### Questionnaires interview

A comprehensive questionnaire interview was offered to all participants. The questionnaire included data in domains of knowledge attitudes and practices related to blood-borne diseases transmitted occupationally, safe injection practices, standard precautions to prevent occupationally transmitted blood borne infections, hepatitis B vaccination in early childhood and in the previous 5 years.

### Blood sampling

Approximately 5–10 ml venous blood samples in plain tubes were taken from each participant and were allowed to clot at room temperature (range 18°C to 20°C). Samples were then centrifuged at 10,000 rpm for 10 min, and the separated sera were aliquoted into two portions and stored at −20°C until transported in ice boxes to the Virus Lab of Abha college of Medicine, where they were grouped by the area of collection and stored in classified boxes in similar conditions as described above.

### Serologic tests for HBV markers

The HBV markers were detected by standard enzyme-immunosorbent assay (ELISA) using commercial kits for anti-HBc IgG, anti-HBs, HBsAg, HBe Ag/anti-HBe and anti-HBc IgM according to manufacturers’ instructions.

Initially, all sera were tested for anti-HBs. Reactive sera were tested for recovery markers following natural infection (anti-HBc-IgG and anti-HBe) to differentiate them from vaccinees. Subsequently, anti-HBc IgG, a marker detectable in the late acute, window, and recovery phases of HBV infection and in chronic virus carriers (both symptomatic and asymptomatic) were tested. HBsAg was tested for all sera, excluding those reactive to anti-HBs (through natural infection or vaccination). Positively reacting sera for HBsAg were further tested for HBe Ag/anti-HBe, anti-HBc IgM to determine the clinical stage of the disease: early acute, acute, window, or possibly chronic carriers who will eventually require a follow-up.

### Testing for HCV

The laboratory diagnosis of HCV infection was performed utilising third-generation ELISA detecting IgG class antibodies against the viral structural protein c-22-3 derived from the genomic core region and three non-structural proteins c-33c, c100-3 and 5-1-1 derived from the NS3 and NS4 regions of the viral genome. A novel ELISA was utilised to confirm the initial immunoassay findings. Equivocal results were further tested by PCR to confirm the results.

### Statistical analysis

Data were coded, validated and analysed using the Statistical Package for the Social Sciences (SPSS), version 13.0 (SPSS Inc., Chicago, IL, USA). The frequency, percentage, arithmetic mean, and mode are used to present the data. A Chi square test was used as the test of significance at a 5% level of significance.

## Results

### Description of the study sample

The present study included 300 HCWs and 300 HS in the Najran region (Table 1). The age of the HCWs ranged from 18 to 61 years with an average of 34.5 ± 10.1 years. There were 81 male HCWs (27.0%) and 219 female HCWs (73.0%). The majority of health care workers were non-Saudis (n = 255, 85%), and the rest were Saudis (n = 45, 15%). The HCWs included 58 physicians (19.3%), 10 dentists (3.3%), 200 nurses (66.7%) and 12 laboratory workers (4%). The majority of HCWs were working in hospitals (n = 223, 74.3%), and the rest worked in primary health care centres (n = 77, 25.7%).

**Table 1:** Description of the study sample of Najran Health Care Workers (NHCWs) and Najran Health Students (NHS)

**Variable**	**NHCW (N = 300)**		**NHS (N = 300)**	
	**Number**	**%**	**Number**	**%**
**Gender:**				
Males	81	27.0	199	66.3
Females	219	73.0	101	33.7
**Place of work:**				
Primary health care	77	25.7	-	-
Hospitals	223	74.3	-	-
**Health college:**				
Medicine	-	-	31	10.3
Dentistry	-	-	21	7.0
Pharmacy	-	-	32	10.7
Medical laboratory	-	-	34	11.2
Radiology health	-	-	38	12.7
Nursing	-	-	44	14.7
Physiotherapy	-	-	59	19.7
Midwifery			41	13.7

The age of HS ranged from 18 to 25 years with an average of 20.9 ± 1.3 years. The study included 199 males and 101 females. Thirty-one per cent (93) were aged 23 years and older (born before the introduction of the compulsory childhood Hepatitis B vaccine in the national programme of immunisation), and the rest of the HS (n = 207, 69.0%) were aged 18 to 22 years (born after the introduction of the vaccine).

### Knowledge, attitudes and practice

Table 2 shows that the majority (99.0%, n = 297) of HCWs were aware of all blood-borne diseases (HIV, HBV, HCV) transmitted occupationally and all procedures that may expose workers to blood-borne diseases transmitted occupationally (87.3%, n = 263). More than half of HCWs are aware of all safe injection practices that may protect them (53.0%, n = 159) and all standard isolation precautions to prevent occupationally transmitted blood-borne infections (72.6%, n = 218). The majority (97.3%, n = 292) of HCWs had a positive attitude towards routinely testing all healthcare workers for Hepatitis B and C and restricting hepatitis B and C-positive health care workers to low-risk procedures (66.7%, n = 200). The majority of HCWs (84.7%, n = 258) had received a hepatitis B vaccination in the previous 5 years.

**Table 2:** Knowledge, attitudes and practice of Najran Health Care Workers (NHCWs) and Najran Health Students (NHS)

**Knowledge, attitudes and practice**	**NHCWs (N = 300)**		**NHS (N = 300)**	
	**No**	**%**	**No**	**%**
Aware of all Blood Borne Disease transmitted occupationally (HIV, HBV, HCV)	297	99.0	105	35.0
Aware of all Procedures that may expose to Blood Borne Disease transmitted occupationally (Injections, Blood sampling, Incisions)	263	87.3	57	17.3
Aware of all Safe Injection Practices (never bend needles, never recap needles and never remove needles after use)	159	53.0	45	15.0
Aware of all Standard Precautions to Prevent Occupationally Transmitted Blood Borne Infections (Hand hygiene, gloves, aprons, safe disposal of sharp and needles).	218	72.6	30	10.0
Positive attitudes towards Testing routinely all healthcare workers for Hepatitis B and C	292	97.3	243	81.0
Positive attitudes towards restricting hepatitis B and C positive health care workers to low risk procedures	200	66.7	180	60.0
Hepatitis B Vaccination in early childhood	158	52.7	87	29.0
Hepatitis B Vaccination in the previous five years	258	84.7	69	23.0

On the other hand, Table 2 revealed poor or insufficient knowledge of the blood borne infections among students. Health students had less knowledge than HCWs. Less than one-third of the students properly identified the blood borne infections (35.0%, n = 105), were aware of all blood-borne diseases (HIV, HBV, HCV) transmitted occupationally, all procedures that may expose to blood-borne diseases transmitted occupationally (17.3%, n = 57), all safe injection practices that may protect them (15.0%, n = 45) and all standard isolation precautions to prevent occupationally transmitted blood-borne infections (10.0%, n = 30). However, the majority (81.0%, n = 243) of HS had a positive attitude towards testing routinely all healthcare workers for Hepatitis B and C and restricting hepatitis B and C-positive health care workers to low risk procedures (60.0%, n = 180). Only 87 (29.0%) HS received hepatitis B vaccination in early childhood, and 69 (23.0%) HS received the vaccine in the previous 5 years.

### Sero-prevalence of hepatitis B markers

Table 3 indicates that one (0.3%) HCW was positive for hepatitis B surface antigen, giving a prevalence of 3.3 per thousand. The positive case was a Saudi female nurse, aged 24 years, working in primary health care centres. Her main work was injection and dressings. Conversely, all HS were found negative for HBsAg, giving a prevalence of zero per cent.

**Table 3:** Hepatitis “B” markers and HCV among Najran Health Care Workers (NHCWs) and Najran Health Students (NHS)

**Laboratory test**	**NHCWs (N = 300) Positive n (%)**	**NHS (N = 300) Positive n (%)**
**Hepatitis “B” Markers**		
HBsAg	1 (0.3)	0 (0.0)
anti-HBs	230 (76.2)	100 (33.3)
anti HBc IgM	1 (0.3)	0 (0)
Anti HBcIgG	25 (8.0)	5 (1.7)
HBe Ag	1 (0.3)	0 (0.0)
Anti HBe	25 (8.0)	5 (1.7)
**HCV**	0 (0.0)	1 (0.3)

Among HCWs, 70 (23.3%) were negative for anti-HBs, indicating that they are not immune and are susceptible to infection. Among HCWs in primary health care centres 67.5% (n = 52) were negative to anti-HBs compared with 8.1% (18) among hospitals HCWs. The difference was significant (χ^2^ = 113.1, P = 0.001). Laboratory workers were the profession most lacking anti-HBs (n = 8, 66.7%) followed by other professions (n = 8, 40.0%) and dentists (n = 3, 30.0%). The lowest were nurses (n = 39, 19.5%) and physicians (n = 12, 20.7%). The difference was significant (χ^2^ = 42.2, P = 0.001).

Among HS, 200 subjects were negative for anti-HBs (66.7%), indicating that they are not immune and are susceptible to infection. One (0.3%) HCW and none of the HS were positive for Anti-HBc IgM. Twenty-five (8.0%) of HCWs and 5 (1.7%) HS were positive for Anti-HBc IgG. One (0.3%) HCW and none of HS were positive for HBe Ag. Twenty-five (8.0%) HCWs and 5 (1.7%) HS were positive for anti-HBe.

### Overall seroprevalence of HBV infection

An overall prevalence of 8.7% was found among HCWs. This prevalence included one acutely infected case (HBsAg and anti-HBc IgM positive) and twenty-five cases who recovered from previous exposures to HBV infection (anti-HBs, anti-HBc IgG and Anti-HBe positive). Similarly, an overall prevalence of 1.7% was found among HS. This prevalence included five cases who recovered from previous exposures to HBV infection (anti-HBs and anti-HBc IgG positive).

### Sero-prevalence of hepatitis C

Four samples of HCWs were found to be equivocal by ELISA and the confirmatory EIA techniques. Using a qualitative PCR technique, all of the equivocal samples were determined to be negative, giving a prevalence of zero per cent. On the other hand, one sample (0.3%) of HS was found to be positive for Hepatitis C by ELISA and the confirmatory EIA. The sample was also found to be positive in the qualitative PCR, giving a prevalence of 3.3 per thousand. The positive case was a male medical laboratory student, aged 19 years.

## Discussion

### Main findings of the study

An overall seroprevalence of HBV of 1.7% was found among the HS. The study revealed a lack of an acute/recent infection of HBV infection. In the study of Al-Ajlan [17], a prevalence of 0.1% of recent HBV infection was found among health colleges students in Saudi Arabia. The striking finding in our study was that approximately two-thirds of Najran health students (n = 200, 66.7%) lack anti-HBs and hence are susceptible to HBV infection. Males (n = 145, 72.9%) were more susceptible than females (n = 55, 54.5%). This gender variation has also been reported by other studies in Saudi Arabia [18].

### What is already known on the topic

The Kingdom of Saudi Arabia introduced mandatory HBV vaccination of all children 22 years ago in 1990 [4], and because nearly all of the Najran health students enrolled in this study were born a few years later, it is expected that virtually all of them had received the mandatory vaccination against HBV in the national EPI. When students were asked about their immunisation history, only 87 (29%) students recalled being vaccinated in early childhood. The remaining seropositives for anti-HBs among Najran students acquired their immunity through either their own initiative for taking the vaccine (n = 69, 23%) or following a natural subclinical infection (n = 5, 1.7%) as documented by the presence of both anti-HBc-IgG and anti-HBe. None of the subclinical infections among students were related to exposure during their practice, history of operations or blood transfusion.

### What this study adds

Although this was a selected group, the low prevalence of acute/recent HBV infections among the susceptible students appears to indicate the effectiveness of HBV vaccination adopted by the Kingdom since 1990 in reducing the circulation of HBV among the general population. The efficacy of the vaccine used, in terms of its long-term protection, has also been well-documented [13]. Nevertheless, this high proportion of susceptible students in Najran health colleges calls for a novel approach regarding their vaccination: the authors believe that students to be enrolled in these colleges should be considered as new recruits to health services in terms of their initial screening for blood-borne infections and vaccination against HBV. The guidelines adopted by the Kingdom of Saudi Arabia in that respect should be strictly implemented and followed [19].

A 19-year-old male medical laboratory science student was infected with HCV giving a seroprevalence of 3.3 per thousand. This finding is higher than that reported by Al-Ajlan [17] where a prevalence of 0.03% of HCV infection was found among male health science students of a similar age group (18 – 21 years vs. 18 – 25 years in this study). Most of the reports of HCV prevalence in Saudi Arabia have been cross-sectional studies among blood donors, giving a range of 0.4% to 1.1% [7,20,21]. In a comprehensive review of the epidemiology of viral hepatitis [22], it was reported that despite the observed decline in HCV prevalence in the last 10 years in Saudi Arabia, HCV disease still constitutes a major health problem in Saudi Arabia.

Among the 300 HCWs, a female Saudi nurse (n = 1, 0.3%) mainly working with dressings and giving injections was subclinically and acutely infected with HBV as documented by the presence in her serum sample of HBsAg, HBeAg and anti-HBc-IgM. She did not report any risk factor included in the questionnaire to account for her infection. Seventy (23.3%) HCWs had no detectable anti-HBs and thus are vulnerable to HBV infection. There were 45 Saudis among these workers, representing 15% of the working force in Najran health centres: Of these Saudis, almost two-thirds (n = 18, 62.2%) had no detectable anti-HBs and thus are prone to HBV infection. This is in contradistinction to the 42 non-Saudis (16.5%) who were seronegative for anti-HBs. Comparing the two groups, the difference was significant (p = 0.001). The reason for this difference is most likely that the majority of the non-Saudi work force receives their HBV vaccine prior to their recruitment. It is interesting to note that among these workers, twice as many males were seronegative compared with females (38.3% vs. 17.8%), thus rendering a higher proportion of males to be prone to HBV infection. This gender variation has already been discussed [18]. Two-thirds (n = 52, 67.5%) of those seronegatives were working in primary health care centres, whereas only 18 (8.1%) worked in hospitals. The difference was significant (p = 0.001). Based on these results, the authors feel that health care workers who are males, Saudis and working in primary health care centres are at a special risk of acquiring HBV infection and therefore should all be screened to determine their state of immunity to HBV and be vaccinated if found seronegative. None of the health care workers displayed any evidence of HCV infection.

The present study reported poor knowledge among health students in Najran University regarding blood-borne diseases transmitted occupationally, safe injection practices, and standard precautions to prevent occupationally transmitted blood-borne infections. Guidelines for standard isolation precautions are mainly developed to prevent transmission of blood and body fluid pathogens to HCWs. Our study revealed a generally poor knowledge and compliance with standard isolation precautions among Najran health students. Similar results were reported among Iranian medical students [23] and among Korean nursing and medical students [24]. Inexperience and lack of knowledge and technical expertise place the health student at potential risk. Education and training in infection control for health students should therefore be initiated at the earliest opportunity. Health awareness at the beginning of the pre-clinical training is helpful for making individuals aware of occupational risks, immunisation policies and the importance of universal standard safety precautions. To combat the risk of occupational exposure among Najran health students, it is mandatory to develop a structured program to raise awareness among students in different health colleges. Similarly, current health college curricula should be upgraded to address these issues early, in particular, blood-borne diseases transmitted occupationally, standard precautions to prevent occupationally transmitted blood-borne infections and safe injection practices.

The present study documented a moderate level of knowledge among HCWs in the Najran region regarding blood-borne diseases transmitted occupationally, safe injection practices, and standard precautions to prevent occupationally transmitted blood-borne infections. Similar low to moderate levels of knowledge have also been reported elsewhere [25-27]. Several studies indicate the fact that incomplete health care provider knowledge of blood transmission may jeopardise the effectiveness of prevention and control programs for HBV and HCV [28-30].

A revision of the current continuing medical education programs for health care workers offered in Najran may be required that mandates that all health care workers should attend infection control courses specific to their clinical terms. The development of a novel continuing medical education and pre-employment awareness program for health care workers is recommended to address blood-borne diseases transmitted occupationally, standard precautions to prevent occupationally transmitted blood borne infections, and safe injection practices.

### Strength and limitations

The strength of the present study is that it addresses a major health problem that confronts health care workers and health students and thus they need to know about the disease and how to protect themselves. Furthermore, the study sends a strong message to the universities in Saudi Arabia to develop and adopt a clear protocol regarding HBV protection prior to the practice of the students. History of vaccination against HBV did not appear a valid indicator of the students’ immunity status.

The study limitations are mainly related to the fact that it was based on self reporting of the candidates and no attempt was made to revise their health records. Similarly, practices were based on the reported responsed of the participants and no attempt was made to directly observe their practices.

## Conclusion

It is mandatory to develop a structured program to raise awareness among HS, and current health colleges’ curricula should be upgraded to address these issues early. The HS should be considered new recruits to health services in terms of their initial screening for blood-borne infections and vaccination against HBV. The development of a novel continuing medical education and pre-employment awareness program for HCWs is recommended to address the following: blood-borne diseases transmitted occupationally, standard precautions to prevent occupationally transmitted blood borne infections, and safe injection practices.

## Competing interests

The authors declare that they have no competing interests.

## Authors’ contributions

AAE and AMA conducted the laboratory analysis. AAM conducted the data analysis. All authors designed, conducted the literature review, interpreted the results, commented on various drafts of the article and wrote the manuscript. All authors approved the final version and accept responsibility for the paper.

## Pre-publication history

The pre-publication history for this paper can be accessed here:

http://www.biomedcentral.com/1471-2458/14/577/prepub
